# The Book World of Medicine and Science

**Published:** 1896-06-27

**Authors:** 


### THE BOOK WORLD OF MEDICINE AND SCIENCE.

BURDETT'S HOSPITALS AND CHARITIES, 1896, BEING THE YEAR BOOK OF PHILANTHROPY. By HENRY C. BURDETT. (London: The Scientific Prass. pp. 856. Price 5s.)

We gladly welcome the new edition of this most useful year book of philanthropy in its various manifestations. It is no small testimony to the dimensions reached by English charity that the demand for such a work as this should enable the publishers to issue so complete a work at such a price. It is not everyone who wants a handbook to the charities of his country, and it shows the immensity of what we may call the business of philanthropy that a sufficient number of people should be so personally and actively concerned in philanthropic effort as to create a demand such as to justify the production of a book of 850 pages, beautifully printed from the cleanest possible type on well pressed paper, containing the latest information concerning hospitals, dispensaries, convalescent homes, nursing institutions, missionary and religious societies, orphanages, homes, and institutions for the blind, the deaf and dumb, the incurable, and the inebriate in England, America, and the colonies, together with a most readable and interesting account of the year's progress in charitable affairs occupying more than 250 pages. It is quite impossible here to mention the many matters dealt with in the latter. We would, however, especially draw the attention of all interested in the development of working-class charity to the very elaborate investigation, detailed in chapter xiii., as to how far the contributions of artizans to certain funds may be looked upon as free gifts—*i.e.*, gifts without any return in benefit of any kind. So far as hospitals are concerned, it is one of the most important questions of the day. To the medical profession probably the most interesting chapter will be found to be that on the misuse of hospitals. On this matter it is said that "the first lesson undoubtedly is that the system of admitting patients to the metropolitan hospitals requires reform and amendment. No one who has studied the question and taken care to ascertain the classes which at present supply patients to the hospitals can doubt that a vast number of them ought not to receive free medical relief." It is maintained that whatever other restrictions may be placed on the admission of patients, those who do ultimately get in, if they can pay, should be made to pay The fact is that the better the internal management of the hospitals the more the well-to-do classes will force themselves in. Self-preservation is the first law of nature, and when people are seriously ill they care but little in what company they journey towards health. They believe that in the hospitals their best hope of relief lies, "and if the hospitals will not permit them to pay for it then they will pocket their feelings and accept free relief." A very short inspection of this book will show that it is much more than a mere hospital directory, there is, in fact, a great deal in the introductory portion which is of permanent interest.

THE PATHOLOGY OF THE CONTRACTED GRANULAR KIDNEY AND THE ASSOCIATED CARDIO-ARTERIAL CHANGES. By SIR GEORGE JOHNSON, M.D., F.R.S. (London: J. and A. Churchill. 1896. Price 4s.)

Sir George Johnson has had the last word. Gull is dead, Sutton is dead, and now, published while Johnson lay on his deathbed, comes this book, which is not only his last word in his great controversy, but is the actual last word on the subject on which it treats. Not, however, that there is much that is new in the volume before us; but it is a re-statement, enforced by more modern experience, of the views so vigorously maintained against all comers in the days of Sir George Johnson's controversy with the authors of the theory of arterio-capillary fibrosis. To those who look on after the fray, the thought is apt to occur that much of the difference arose from an unwillingness to depart a hair's breadth from what appeared a neat and admirable explanation of the observed phenomena. Even in these last words the great exponent of the stop-cock theory does not budge from the position he took up so many years before, and not only declines to accept the conclusions of those who differ from him, but, as of old, protests against their interpretations of their own specimens. Great, however, as was the credit due to Sir George Johnson for his observations on the cardio-vascular charges which take place in connection with contracted kidney, it by no means follows, even if we grant to the full the muscularity of the thickening observed in the arterioles, that the stop-cock action of these arterioles operated in the way that he supposed. But, as was seen by Gull and Sutton, the cardio-vascular changes in chronic Bright's disease involved something more than the contradictory see-saw between heart and arterioles which Johnson described. That something more was a gradually developing arterio-capillary fibrosis; but it does not even yet seem entirely clear what is the relation between this sclerotic change which undoubtedly often exists as a final product, and the muscular hypertrophy which equally undoubtedly can be demonstrated in certain cases. Needless to say, the case for the stop-cock theory—for his work in regard to which Sir George Johnson will probably be longest remembered—is very well put before his readers in these his last words. The second portion of the book deals with what is first in the title, viz., the pathological changes in the contracted granular kidney, and aims at showing that the term interstitial nephritis is inappropriate, and that the pathological changes are primarily and essentially intra-tubular.

